# The Spectrum of Autoimmune Liver Disorders, Clinical Presentation, and Autoantibodies in Patients From a Tertiary Care Center in Pakistan

**DOI:** 10.7759/cureus.19789

**Published:** 2021-11-21

**Authors:** Zaigham Abbas, Muhammad Asim, Alina Saeed, Basit Siddiqui, Minaam Abbas

**Affiliations:** 1 Gastroenterology and Hepatology, Dr. Ziauddin University Hospital, Karachi, PAK; 2 Gastroenterology, Dr. Ziauddin University Hospital, Karachi, PAK; 3 Internal Medicine, Dr. Ziauddin University Hospital, Karachi, PAK; 4 Gastroenterology, Fazaia Ruth Pfau Medical College, Karachi, Karachi, PAK; 5 Internal Medicine, School of Clinical Medicine, University of Cambridge, Cambridge, GBR

**Keywords:** autoantibodies, cirrhosis, overlap syndrome, primary biliary cholangitis, autoimmune hepatitis

## Abstract

Background

The autoimmune illnesses that affect the liver include autoimmune hepatitis (AIH), primary biliary cholangitis (PBC), primary sclerosing cholangitis (PSC), and overlap syndrome. In our patients, we aimed to address the complete spectrum of autoimmune liver disorders, clinical presentation, and autoantibodies.

Methods

The study included all the patients diagnosed with autoimmune liver disorder irrespective of age, gender, and ethnic background presented at the liver clinic of the hospital in the last two years. The diagnosis was based on characteristic clinical and laboratory findings, the presence of one or more characteristic autoantibodies, and/or histological abnormalities. The diagnosis of AIH was further validated by revised International AIH Group criteria using a scoring calculator. The diagnostic criteria for PBC required the presence of chronic elevation of alkaline phosphatase (ALP) with positive antimitochondrial antibody (AMA) or positive PBC-specific anti-nuclear antibodies (ANA) (sp-100, gp-210) tests and/or compatible histology. The patients of AIH-PBC overlap syndrome fulfilled the criteria for AIH in the setting of PBC. Patients having liver involvement in other autoimmune disorders were included in the study.

Results

The total number of patients was 124; 83 (67%) were females; mean age ± standard error of mean (SEM) was 44.97 ± 1.47 years with a range of 09-84 years. Type-1 AIH was seen in 68 (54.8%) patients, type-2 AIH in 10 (8.1%) patients, PBC in 22 (17.7%) patients, overlap of PBC with AIH in 10 (8.1%) patients, IgG4 disease in four (3.2%) patients, psoriasis-specific immune hepatitis in four (3.2%) patients, celiac disease-related hepatitis in three (2.4%) patients, sarcoidosis in two (1.6%) patients, and ichthyosis-associated hepatitis in one (0.8%) patient. There was a high prevalence of cirrhosis (50%) at the time of presentation; 19% of patients had decompensated liver disease. ANA was positive in 52/68 cases of AIH type-1, but anti-smooth muscle antibody (ASMA) was reactive only in nine cases and anti-soluble liver antigen (SLA) in five cases. There was no female preponderance in type-2 AIH (M:F = 6:4). AMA was reactive in 25 (78%) cases of PBC and overlap syndrome. Antibodies prevalent in PBC (AMA-M2, AMA-M2-3E, sp-100, gp-210, anti-Ro52) were also seen in some cases of AIH, though they did not fulfill the criteria of the overlap syndrome.

Conclusion

There is an unmet need for the early diagnosis of autoimmune liver diseases and the initiation of appropriate management to prevent complications.

## Introduction

Autoimmune liver diseases are conditions associated with immune-mediated injury of hepatocytes or bile ducts that include autoimmune hepatitis (AIH), primary biliary cholangitis (PBC), and primary sclerosing cholangitis (PSC), immunoglobulin G4-associated cholangitis, and their poorly characterized overlapping syndromes [1]. Autoimmune liver disease is considered a less common cause of chronic liver disease in Pakistan. Causal factors are incompletely understood, but immunogenetic susceptibility and environmental triggers are believed to generate an unregulated T-cell response against hepatocyte and cholangiocyte autoantigens. A potpourri of risk factors and their interplay including gender, ethnicity, bacteria and viruses such as hepatitis viruses, drugs, toxins and xenobiotics, associated autoimmune diseases, immune and non-immune genes, genes of the MHC locus, and epigenetics decides the pattern of the autoimmune process in the liver and which disease phenotype may progress [2,3].

Characteristics of AIH include female predilection, elevations of aminotransferases, non-specific or organ-specific autoantibodies, increased levels of gamma globulin IgG, interface hepatitis on liver biopsy, and response to the immunosuppressive treatment [4]. It affects women 3.6 times more commonly than men. It is a progressive liver disease that afflicts children and adults of all ethnicities and races [5,6]. Up to 80% of patients present with chronic hepatitis, while 33% have cirrhosis, indicating a propensity for insidious progression. However, the spectrum of presentations of AIH includes very rarely occurring acute liver failure, infrequent acute hepatitis, and asymptomatic patients [7]. Cirrhosis confers risks of complications of portal hypertension, liver failure, and hepatocellular carcinoma (HCC). Gamma-glutamyl transferase (GGT) and alkaline phosphatase (ALP) levels are elevated in PBC and PSC, while IgM is elevated only in PBC patients. Associations with other autoimmune diseases are common.

The prevalence of autoimmune liver disorders is low in many Asian countries including Pakistan [8]. Perhaps, dietary and environmental factors play some protective role and decrease the susceptibility and vulnerability to develop autoimmune liver disease. In the migrant population from South Asia settled in the United Kingdom, PBC has a higher prevalence than their countries of origin [9]. Another reason for the low prevalence of autoimmune liver disease in our country may be the lack of knowledge and awareness among clinicians, especially when viral hepatitis is the diagnosis in most of the patients and many patients with AIH are detected at an advanced stage [10]. Studies addressing the clinical profile of PBC from Pakistan are rare. This study aimed to determine the nature and the clinical profile of the autoimmune liver disorders presenting alone or in association with other autoimmune disorders.

## Materials and methods

The study included all the patients diagnosed with autoimmune liver disorder irrespective of age, gender, and ethnic background presented at the liver clinic of the hospital in the last two years. The diagnosis of AIH was based on characteristic clinical and laboratory findings (elevated levels of serum aspartate aminotransferase [AST] and alanine aminotransferase [ALT] as well as increased serum IgG concentration) and the presence of one or more characteristic autoantibodies and/or histological abnormalities (interface hepatitis). The antibodies used for the diagnosis of type-1 AIH were anti-nuclear antibody (ANA), ASMA, and anti-soluble liver antigen (SLA). Antibodies for type-2 AIH were anti-liver kidney microsomal antibody (anti-LKM-1) and anti-liver cytosol type-1 (anti-LC-1) antibodies. The diagnosis of these cases was further validated by revised International AIH Group criteria using a scoring calculator [11]. A score of > 15 was taken as confirmed AIH and ≥ 10 as probable AIH. Other causes of chronic liver disease (CLD) were ruled out by clinical examination, imaging, biochemical tests, relevant serology, and histopathology of the liver. The diagnostic criteria for PBC required the presence of chronic elevation of ALP for more than six months with positive AMA or positive PBC-specific ANA (sp-100, gp-210) tests and/or compatible histology (especially lymphocytic cholangitis of small- to medium-size bile ducts and granulomas) [12]. The presence of ANA, anti-smooth muscle antibody (ASMA), and AMA was tested by the indirect immunofluorescent method. The rest of the autoimmune liver disease antibody profile was checked by the immunoblot method.

The patients of AIH-PBC overlap syndrome fulfilled the criteria for AIH in the setting of PBC: serum ALT level ≥ five-fold upper limit of normal (ULN) and serum IgG level ≥ 1.5-2-fold ULN or presence of characteristic AIH antibodies and the presence of interface hepatitis [12-14]. Biopsy was done in 50 cases where the diagnosis of AIH or PBC was not clear or overlap syndrome was suspected.

Exclusion criteria were patients with a score of less than 10 in the International AIH Group criteria scoring calculator, viral infection, metabolic disorder, drug intake, individuals with a history of hepatectomy for HCC, and liver transplant for HCC. The demographic, clinical, and laboratory data from each patient were gathered and analyzed. Ziauddin Ethics Review Committee (ZU-ERC) approved the analysis of data (Ref: 1320819ZAGE).

Statistical analysis

Statistical interpretation of data was performed using the computerized software program Statistical Package for the Social Sciences (SPSS) version 22.0 (IBM Corp., Armonk, NY). Results were expressed as mean ± standard error of mean (SEM) for continuous variables (e.g., age) and number (percentage) for categorical data (e.g., gender, etc.). The data were analyzed using the Kruskal-Wallis test, Mann-Whitney U test, Pearson chi-square test, or Fisher's exact test where appropriate. A p-value of <0.05 was considered statistically significant. All the p-values were two-sided.

## Results

**Figure 1:**
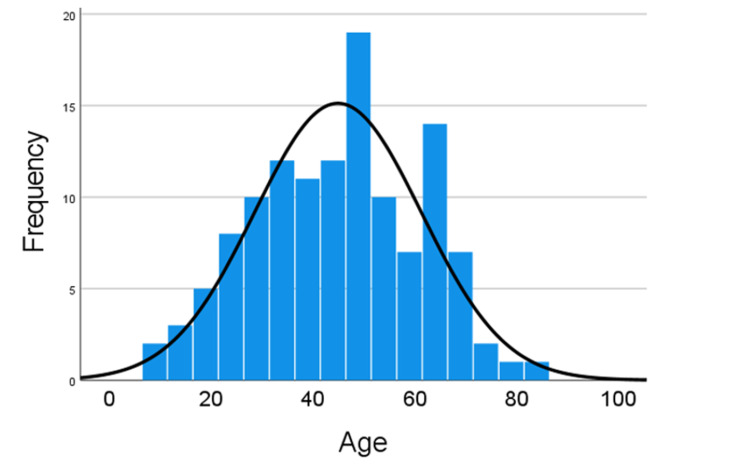
Age frequency of the study patients

The total number of patients with autoimmune liver disorders identified was 124; females were 83 (67%). The mean age was 44.97 ± 1.47 years with a range of 09-84 years. Most of the patients were in their fourth or fifth decade (Figure 1).

Type 1 AIH was present in 68 (54.8%) patients, type 2 AIH in 10 (8.1%), and PBC in 22 (17.7%). The overlap of PBC with AIH was observed in another 10 (8.1%) cases. Miscellaneous causes and associated autoimmune disorders with liver disease are mentioned in Table 1.

**Table 1:** Clinical presentation of autoimmune disorders

Clinical presentation	
Gender: male/female	41/83
Age: years, mean ± Std. error of mean (range)	44.97 ± 1.47 (09-84)
Diagnosis	N (%)
Autoimmune hepatitis type 1	68 (54.8)
Autoimmune hepatitis type 2	10 (8.1)
Primary biliary cholangitis	22 (17.7)
Overlap hepatitis	10 (8.1)
IgG4-related disease	4 (3.2)
Psoriasis-specific immune hepatitis	4 (3.2)
Celiac disease-related hepatitis	3 (2.4)
Sarcoidosis	2 (1.6)
Ichthyosis-associated hepatitis	1 (0.8)
Cirrhosis	
Cirrhosis	62 (50.0)
Child A	39 (31.5)
Child B	16 (12.9)
Child C	07 (5.6)
Esophageal varices	28 (22.5)
Associated diseases	
Diabetes mellitus	33 (26.6)
Hypothyroid	7 (5.6)
Systemic lupus erythematosus	4 (3.2)
Autoimmune hemolytic anemia	1 (0.8)
Idiopathic thrombocytopenic purpura	1 (0.8)
Hepatocellular carcinoma	2 (1.6)
Morphea	1 (0.8)
Recurrent miscarriages	1 (0.8)
Polycystic ovary	2 (1.6)
Focal nodular hyperplasia	1 (0.8)
Amenorrhoea	1 (0.8)

A comparison of clinical and laboratory parameters of AIH, PBC, and the overlap syndrome is mentioned in Table 2.

**Table 2:** Comparison of clinical and laboratory parameters of AIH, PBC, and the overlap syndrome AIH, Autoimmune hepatitis; PBC, primary biliary cholangitis; ALT, alanine aminotransferase; AST, aspartate aminotransferase. Values are mean ± standard error of mean. P-values were identified by independent-samples Kruskal-Wallis test and Pearson chi-square test or Fisher’s exact test.

	AIH (n = 78)	PBC (n = 22)	Overlap (n = 10)	P-value
Age: years ± SEM	45.31 ± 2.09	44.59 ± 2.53	37.60 ± 2.71	0.252
Age: median (range)	50 (11-84)	45 (22-63)	36 (23-48)	
Male:female	32:46	1:21	0:10	<0.001
Weight (kg)	61.42 ± 1.45	59.41 ± 1.94	61.33 ± 5.76	0.722
Presenting complaints				
Fatigue	43	8	2	0.052
Icterus	20	3	4	0.252
Itching	15	5	4	0.324
Abdominal pain	24	4	1	0.232
Abdominal distension	11	4	1	0.814
Examination findings				
Edema	18	6	0	0.197
Shifting dullness/ascites	19	3	0	0.136
Palpable liver	21	8	5	0.387
Palpable spleen	19	3	0	0.136
No cirrhosis	35	10	6	0.855
Child A cirrhosis	26	8	2	-
Child B cirrhosis	12	2	2	-
Child C cirrhosis	5	2	0	-
Lab parameters				
Hemoglobin [g/dl]	12.02 ± 0.22	11.06 ± 0.38	10.24 ± 0.36	0.005 (AIH-overlap = 0.004, PBC-AIH = 0.044)
Total leucocyte count [x 10^9^]	7.39 ± 0.33	6.95 ± 0.71	6.87 ± 0.76	0.697
Platelets [x 10^9^]	184.5 ± 10.8	203.9 ± 27.1	272.2 ± 59.1	0.302
International normalization ratio	1.26 ± 0.04	1.26 ± 0.04	1.53 ± 0.44	0.821
Bilirubin [mg/dL]	3.42 ± 0.69	3.60 ± 1.39	2.13 ± 0.71	0.988
Direct bilirubin	2.25 ± 0.50	7.81 ± 5.32	1.64 ± 0.63	0.523
ALT [IU/mL]	148.3 ± 24.1	91.6 ± 16.6	147.2 ± 27.0	0.158
AST [IU/mL]	132.9 ± 21.8	89.32 ± 16.2	124.60 ± 19.1	0.149
Gamma-glutamyl transferase [IU/mL]	150.0 ± 20.6	345.4 ± 82.5	239.80 ± 72.9	0.001 (AIH-PBC < 0.001, AIH-overlap = 0.029)
Alkaline phosphatase [IU/mL]	183.5 ± 16.9	329.7 ± 35.3	389.7 ± 125.1	<0.001 (AIH-PBC < 0.001 AIH-overlap = 0.035)
Albumin [g/dL]	3.41 ± 0.07	3.44 ± 0.12	3.70 ± 0.18	0.390
Globulin [g/dL]	4.32 ± 0.49	3.98 ± 0.19	3.41 ± 0.16	0.238
Creatinine [mg/dl]	0.92 ± 0.09	0.78 ± 0.04	0.93 ± 0.06	0.692

Patients with type-2 AIH were younger than those with type-1 AIH; the mean age of AIH-1 was 47.33 ± 2.19 and that of AIH-II was 2,33.00 ± 5.34 years (p = 0.018). Type-2 AIH comprised 13% of cases of AIH. There was no significant difference in the male to female (M:F) ratio in types 1 and 2 AIH (26:42 vs 6:4).

Comparing AIH with PBC and overlap syndrome, there was a significant difference in the hemoglobin, GGT, and ALP levels. Half of the patients suffering from autoimmune disorders had cirrhosis; 55% (43/78) of AIH had cirrhosis at the time of diagnosis, and 50% (16/32) of patients with PBC with or without the overlap syndrome had cirrhosis. We have four patients (M:F = 2:2) of IgG4 disease with disturbed liver function tests (LFTs), elevated transaminases, GGT, and ALP. Two male patients had cirrhosis, and there was evidence of ductal involvement on magnetic resonance cholangiopancreatography (MRCP) in one case. Other autoimmune-related liver disorders included celiac disease-related hepatitis, psoriasis-specific immune hepatitis, sarcoidosis, and ichthyosis-associated hepatitis. The spectrum of autoimmune antibodies in these patents is mentioned in Table 3.

**Table 3:** The spectrum of autoimmune antibodies in patients with autoimmune liver disease AIH, Autoimmune hepatitis; PBC, primary biliary cholangitis.

	AIH1 (n = 68)	AIH2 (n = 10)	PBC (n = 22)	Overlap (n = 10)
Anti-nuclear antibody (ANA)	52	2	7	6
Anti-smooth muscle antibody (ASMA)	9	0	0	0
Antimitochondrial antibody (AMA)	3	0	17	8
Antimitochondrial antibody-M2 (AMA-M2)	5	1	13	9
AMA M2-3E	4	1	13	8
Liver-kidney microsomal antibody (LKM-1)	0	9	0	1
Anti-soluble liver antigen (SLA)	5	0	0	2
Anti-liver cytosol type 1 (LC-1)	0	6	0	0
Gp210	3	2	3	1
Sp100	2	0	5	3
PML	2	1	6	5
Anti-Ro-52	4	2	9	6
Perinuclear anti-nuclear neutrophil cytoplasmic antibodies (P-ANCA)	1	1	0	0

ANA was positive in 52/68 cases, ASMA was positive in only nine cases, and anti-SLA was positive in five cases of type-1 AIH. AMA was reactive in 25/32 cases of PBC and overlap syndrome. Antibodies prevalent in PBC (AMA-M2, AMA-M2-3E, sp-100, gp-210, anti-Ro52) were also seen in some cases of AIH, though they did not fulfill the criteria of the overlap syndrome.

## Discussion

Since few population-based studies of autoimmune liver diseases have been performed in the Asia-Pacific region, epidemiological details including the prevalence and incidence of these entities remain unclear. In the absence of diagnostic biomarkers, AIH is divided according to their signature autoantibodies into type-1 AIH with ANA, ASMA, and SLA antibodies and type-2 AIH with anti-LKM1 or anti-LC1 antibodies. Diagnostic criteria can help determine the probability of AIH, but they are less friendly. These criteria are based on clinical, biochemical, serological, and histological features and responses to empiric immunosuppression [11,15]. We used more simplified criteria for inclusion but double-checked with the validated criteria.

AIH can often be strongly suspected based upon clinical and laboratory features, and thus a liver biopsy may not always be necessary for patients with typical findings on noninvasive testing. We had to opt for liver biopsy in 50 (40.3%) patients when serology was not conclusive or when suspecting an overlap syndrome. We excluded other conditions that can cause chronic hepatitis (viruses, alcohol, drugs, and metabolic causes). In addition to conventional autoantibodies (anti-ANA, SMA, AMA, and LKM-1), we tested our patients for additional autoantibodies as mentioned in Table 3.

A study from Pakistan reviewed 55 patients diagnosed with autoimmune hepatitis (AIH) in the last 10 years [16]. Thirty-six (62.0%) patients had type-1 AIH, 10 (17.2%) had type 2, and the remaining 12 were seronegative with biopsy-proven AIH. Only conventional antibodies were checked, and the extended autoimmune liver panel was not performed. Forty-nine patients (84.4%) had cirrhosis diagnosed on clinical features or biopsy. In the present study, half of the patients suffering from autoimmune disorders had cirrhosis, while 55% (43/78) of AIH had cirrhosis at the time of diagnosis. The higher rate of cirrhosis than what has been described from Europe may be due to the impact of ethnicity on the natural history of autoimmune hepatitis [17]. It may also indicate that many of these patients had a prolonged subclinical phase that could have been detected earlier if some screening liver function testing had become a routine practice.

AIH appears to be increasing in South Asia [18]. The fact whether this is due to ascertainment bias caused by increasing recognition of AIH by clinicians or whether this reflects a true increase in AIH remains unknown. In our series, ANA was positive in 52/68 cases of AIH type-1, but ASMA was reactive only in nine cases and anti-SLA in five cases. The prevalence of ASMA was much lower than described in other studies [19]. We should not miss the diagnosis of autoimmune liver disease in the absence of classical antibodies [20]. Extended autoimmune liver profile and liver biopsy are helpful in such cases. Some of our patients with AIH shared some antibodies more characteristic of PBC, but they could not be characterized as the overlap syndrome (Table 3). The presence of these antibodies may point to some biliary duct damage. But it should not alter the diagnosis or treatment of AIH as these patients lack typical features of PBC, and they respond well to the corticosteroid therapy [21,22].

Some of our AIH patients had other associated autoimmune diseases as described in the literature [23], and one had developed HCC. Although our patients with type-2 AIH were younger than type-1, not all of them presented in the adolescent age, and there was no female preponderance as described in the Western studies [5]. Moreover, they comprised 13% of AIH cases, more common than in the United States [19]. We did not observe the bimodal age of presentation in AIH, described in the Western literature [24]. This may be related to the small sample size or different disease behaviors. 

A female predominance is seen in patients with autoimmune disorders. In our series, 83 (67%) patients were females, and all the patients with PBC or overlap syndrome were females (31/32) except one. PBC is a progressive chronic cholestatic liver disease that afflicts adults. PBC has the highest female to male sex ratio (10:1) of the autoimmune diseases and is commonly associated with extrahepatic autoimmune diseases [12,25]. The pathology of PBC, which involves the destruction of small to medium caliber interlobular bile ducts, results in progressive bile duct loss (ductopenia), and the obstruction of bile flow promotes deleterious hepatocellular cholestasis. As in AIH, insidious progression can lead to cirrhosis before the diagnosis is made. In our series, 16 (50%) patients with PBC with or without the overlap syndrome had cirrhosis. Our study is the first to report the PBC case series from Pakistan. The reasons for the lack of data may be less awareness about the existence of PBC, limited access to additional antibody testing in the absence of AMA, a high proportion of asymptomatic patients, and the insidiously slow progression of PBC.

The spectrum of the overlap in patients of AIH and PBC may be immune-serologic overlap, e.g., positive ANA, ASMA, and raised IgG in conjunction with AMA and other associated autoantibodies, or a biochemical overlap of transaminases > 5 ULN in patients with PBC, or histologic overlap with lymphoplasmacytic infiltrate or interphase hepatitis in patients with bile duct lesions characteristics of PBC [26]. However, some of the antibodies related to PBC may be reactive in AIH as mentioned before. Recognition of overlap variant is important as the patients of AIH may benefit from ursodeoxycholic acid if they have associated features of PBC. Similarly, patients with predominant PBC may need steroids or immunosuppressive therapy if ALT is much elevated and interphase hepatitis is present [13,27]. We did not have patients of sclerosing cholangitis or overlap of AIH with sclerosing cholangitis in our series.

Immunoglobulin G4-related autoimmune liver disease is now being increasingly recognized [28]. We had four patients (M:F = 2:2) of IgG4-associated AIH. They presented with elevated transaminases, GGT, and ALP. Two male patients had cirrhosis, and there was evidence of ductal involvement on MRCP in one case. Four patients with psoriasis and one patient with ichthyosis had elevated liver-related enzymes with negative autoimmune liver serology. Patients with psoriasis were non-cirrhotic, while the patient with ichthyosis had cirrhosis. Two patients with psoriasis underwent liver biopsy; both had plasma cell infiltration, one patient with lobulitis and another with interphase hepatitis and rosette formation and cholestasis. The association of cutaneous diseases with autoimmune liver disease has been documented but is less well described [29]. Two patients with sarcoidosis had hepatic involvement. They had elevated bilirubin and GGT. Celiac disease-related hepatitis was noticed in three (2.4%) cases. There is a role of the gluten-free diet in reversing liver dysfunction in these patients, and clinicians should consider screening for CD in patients with AIH with persistent elevation of liver enzymes despite immunosuppressant treatment [30].

## Conclusions

The majority of our autoimmune liver disorders patients were in the fourth to fifth decade of their life. A high prevalence of cirrhosis (50%) was there at the time of presentation. About one-fifth of our patients (19%) had decompensated liver disease. Type-I AIH was more prevalent in our patients as compared to type-II AIH and PBC. There was low positivity of ASMA in type-1 autoimmune hepatitis (AIH) patients. Antibodies prevalent in PBC were seen in some cases of AIH, even without overlap syndrome. No female preponderance was seen in type-2 AIH. An extended antibody profile helped to categorize the patients. One-quarter of patients had associated diabetes, and some patients had other associated autoimmune disorders. The delay in the referral and diagnosis may be due to a lack of clinical suspicion of autoimmune liver disease as a cause of CLD. These findings indicate an unmet need for earlier diagnosis, initiation of appropriate treatment, and supportive management to prevent complications, and the need for a liver transplant.
